# High Levels of Serum Ubiquitin and Proteasome in a Case of HLA-B27 Uveitis

**DOI:** 10.3390/ijms18030505

**Published:** 2017-02-26

**Authors:** Settimio Rossi, Carlo Gesualdo, Rosa Maisto, Maria Consiglia Trotta, Nadia Di Carluccio, Annalisa Brigida, Valentina Di Iorio, Francesco Testa, Francesca Simonelli, Michele D’Amico, Clara Di Filippo

**Affiliations:** 1 Eye Clinic of Multidisciplinary Department of Medical, Surgical and Dental Sciences Department, Second University of Naples, Naples 80131, Italy; settimio.rossi@unina2.it (S.R.); carlogesualdo1981@libero.it (C.G.); valedior1976@gmail.com (V.D.I.); francesco.testa@unina2.it (F.T.);francesca.simonelli@unina2.it (F.S.); 2 Department of Experimental Medicine, Section of Pharmacology, Second University of Naples, Naples 80138, Italy; rosa.maisto@unina2.it (R.M.); mariaconsiglia.trotta2@unina2.it (M.C.T.); nadine1988@hotmail.it (N.D.C.); brigida.annalisa@gmail.com (A.B.); clara.difilippo@unina2.it (C.D.F.)

**Keywords:** autoimmune uveitis, ubiquitin, proteasome, prednisone

## Abstract

In this paper, the authors describe a case of high serum levels of ubiquitin and proteasome in a woman under an acute attack of autoimmune uveitis. The woman was 52 years old, diagnosed as positive for the Human leukocyte antigen-B27 gene, and came to our observation in January 2013 claiming a severe uveitis attack that involved the right eye. During the acute attack of uveitis, this woman had normal serum biochemical parameters but higher levels of serum ubiquitin and proteasome 20S subunit, with respect to a healthy volunteer matched for age and sex. These levels correlated well with the clinical score attributed to uveitis. After the patient was admitted to therapy, she received oral prednisone in a de-escalation protocol (doses from 50 to 5 mg/day) for four weeks. Following this therapy, she had an expected reduction of clinical signs and score for uveitis, but concomitantly she had a reduction of the serum levels of ubiquitin, poliubiquitinated proteins (MAb-FK1) and proteasome 20S activity. Therefore, a role for ubiquitin and proteasome in the development of human autoimmune uveitis has been hypothesized.

## 1. Introduction

The ubiquitin–proteasome is a nonlysosomal intracellular protein degradation complex in eucaryotic cells, and is activated by oxidative stress and inflammatory stimuli. The ubiquitin–proteasome pathway plays important roles in many cellular functions, such as protein quality control, cell cycle control and signal transduction. The consequences of the improper cell cycle and signal transduction include defects in ocular development, wound healing, angiogenesis or inflammatory responses. Here, we report a clinical case of a woman suffering from autoimmune uveitis who showed concomitant high levels of serum ubiquitin and proteasome that were almost normalized following oral treatment with prednisone, a synthetic glucocorticoid.

## 2. Case Presentation

The patient was 52 years old and was diagnosed as positive for the Human leukocyte antigen-B27 (*HLA-B27*) gene and negative for ankylosing spondylitis and reactive arthritis as evidenced by the X-ray (RX) and Nuclear magnetic resonance (NMR). She had no psoriasis or inflammatory bowel disease. During the last three years, her clinical history comprised severe attacks of uveitis among other minor symptoms. She came to our observation in January 2013 claiming 3–4 uveitis attacks every year. When she was admitted to our observation, she was under acute attack. This involved the right eye and was characterized by photophobia, blurred vision, visual acuity 3–4/10, floaters, local irido-lens synechiae, and inflammation of the vitreous. The ocular fundus was normal without vasculitis and oedema, but presented slight lifting of the optical disk in the right eye. The clinical signs of uveitis were immediately converted in Standard Uveitis Nomenclature (SUN) for anterior chamber cells and flare: from 0 to 4+; 0= eye at rest, no cells in the field, no flare; 0.5+/1+ = eye redness with perikeratic injection, 1–15 inflammatory cells within the anterior chamber, faint flare, ocular fundus healthy; 2+ = 16–25 inflammatory cells within the anterior chamber, moderate flare; 3+ = 26–50 cells within the chamber, marked flare, rare anterior or posterior synechiae and vitreous cells; 4+ = >50 cells within the anterior chamber, intense flare, anterior or posterior synechiae, presence of fibrinoid exudation in the pupillary area and miosis; vitreitis. Uveitis was considered positive when the score assigned was >1+. According to this classification, the patient was scored 3± when she came to our observation (week 0).

**Figure 1:**
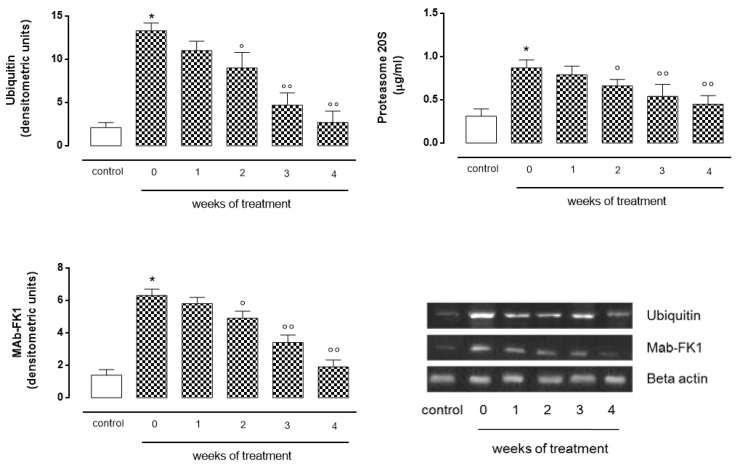
After different weeks of treatment with oral prednisone, levels of serum proteasome 20S were evaluated by ELISA test (Proteasome Elisa kit BML-PW057-0001, Enzo Life Science, Rome, Italy), while levels of ubiquitin and MAb-FK1 were evaluated by Western blotting with specific primary antibodies (anti-ubiquitin sc-8017, Santa Cruz Biotechnology, Heidelberg, Germany; anti-FK1 BML-PW8805-0500, Enzo Life Science, Rome, Italy) and anti-mouse secondary antibody (sc-2005, Santa Cruz Biotechnology, Heidelberg, Germany). Week 0 represents the acute attack of uveitis and the initiationof the prednisone therapy. Values are the means ± S.E.M. of three measurements for each time point. * *p* < 0.01 vs. control; ^ο^ *p* < 0.05 vs. week 0; ^οο^ *p* < 0.01 vs. week 0.

Interestingly, at the acute attack of uveitis (week 0), there were higher levels of serum ubiquitin, polyubiquitinated proteins and proteasome 20S with respect to a healthy volunteer (control) matched for age and sex (week 0, Figure 1), as evaluated on a blood sample, routinely withdrawn at the admission by using Western blotting and specific anti-ubiquitin, anti FK1 antibodies and by Enzyme-linked immunosorbent assay (ELISA). The levels of ubiquitin and MAb to FK1 in the control were 16% and 22% of those measured in the uveitic patient at week 0, respectively. The proteasome levels in the healthy control were 36% of those measured in the patient at week 0. These high levels correlated well with the clinical score attributed to uveitis (*r^2^* = 0.98345). Following medical evaluation, the patient started therapy for uveitis with steroids in a de-escalation protocol. The patient took oral prednisone (two tablets of 25 mg/day) for 7 days. After this first week, the patient received one and a half tablets of 25 mg/day for another week. At the end of the second week, she was visited and the following parameters were registered: visual acuity right eye 7/10; 11–20 inflammatory cells (2+) within the anterior chamber; no posterior synechiae or fibrin in the anterior chamber, moderate flare, unchanged ocular fundus.

**Figure 2:**
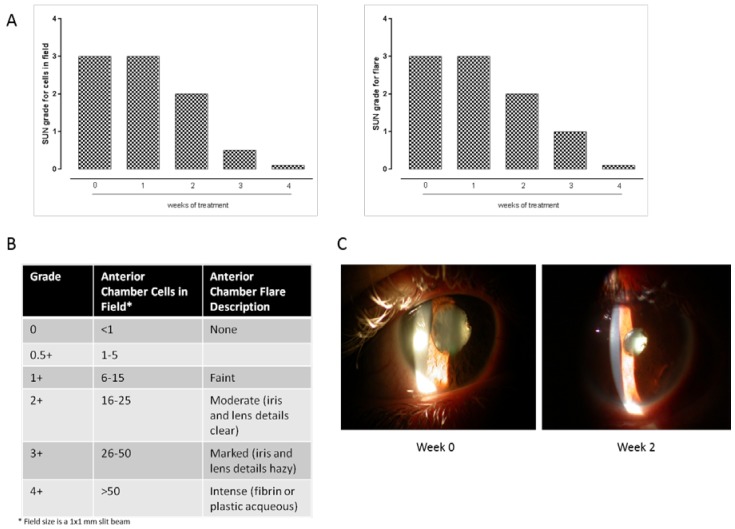
(**A**,**B**) Standardization of Uveitis Nomenclature (SUN) grading scheme attributed to the severity of uveitis during the 4 weeks of treatment with prednisone. The observations refer to the right eye and were made by a blinded ophthalmologist aware of the treatment; (**C**) Anterior segment photos at week 0 and 2.

Concomitantly with the clinical evaluation, after the first two weeks of prednisone treatment, all the common serum parameters were unaltered with respect to the initiation of the therapy but there was a strong and significant (*p* < 0.05 vs. week 0) reduction of the levels of ubiquitin (−32%), poliubiquitinated proteins as evidenced by the FK1 marker (−22% vs. week 0), and proteasome 20S (−24%) (Figure 1) coincident with a reduction of clinical signs and score for uveitis (Figure 2). So, the medical prescription was to continue the oral therapy with one tablet of 25 mg/day for 7 days. At the beginning of the fourth week, this unique patient received three tablets of 5 mg/day for 7 days, and at the end the parameters were as follows: visual acuity right eye 9/10; rare cells (0.5+), no synechiae in the anterior chamber, faint flare, absence of vitreitis, unchanged ocular fundus; the levels of ubiquitin, poliubiquitinated proteins and proteasome 20S decreased up to values of 65%, 38% and 46% respectively, as depicted in Figure 1. The patient continued with two tablets of 5 mg each/day for 7 days and finally 5 mg prednisone (one tablet) for 1 more week until the medical report was as follows: right eye at rest with no cells in the field (0+); visual acuity 10/10; cells absent; no synechiae; no vitreitis; then, coincident with a reduction of clinical signs (Figure 2), a further evident reduction of the three factors was monitored. Values were almost superimposable to those recorded in the healthy volunteer (Figure 1). No adverse event due to the reduction in ubiquitin proteasome activity was noticed during the entire observation.

Written informed consent was obtained from the patient for publication of this case report and any accompanying images.

## 3. Discussion

The ubiquitin–proteasome is a nonlysosomal intracellular protein degradation complex in eucaryotic cells, and is activated by oxidative stress and inflammatory stimuli [1]. Overall, it represents a key regulator of immuno-inflammatory processes leading to tissue derangement. Specifically, it is involved in the development of ocular damage following experimental endotoxic uveitis (EIU) through the activation of the Nuclear Factor κ-light-chain-enhancer of activated B cells (NF-κB,) which, in turn, regulates the expression of the associated inflammatory genes [2]. In line with this, the data presented here indicate, for the first time, that there is an increase of serum levels of ubiquitin, proteasome and their activity during an acute attack of autoimmune uveitis. This high expression is possibly responsible for the ocular damage and the associated high clinical score attributed to the uveitic patient.

Interestingly, the ubiquitin–proteasome levels and clinical score of uveitis were decreased after four weeks of hospitalization and treatment with oral prednisone. We do not know whether it was the steroid decreasing the measured serum levels of the two factors we looked at, or just the time course of uveitis. Notoriously, acute anterior uveitis, even recurrent, tends to be self-limited for a given episode and so a larger study including a larger number of patients is a future aim. It is worth noting, however, that a pioneering study by Hwee and coworkers published in the American Journal of Physiology in 2011 [3] reported a reduction of the proteasome expression and activity in mice heart following oral treatment with the synthetic steroid dexamethasone. In the present case, therefore, we wanted to provide proof of principle that even further insights are needed, especially considering that the mainstay treatment of human autoimmune uveitis tends to inhibit the immune response by systemic agents [4], anti-interleukin-2 (IL-2) receptor antibody [5], α Interferon [6] (IFN-α), or drugs that inhibit the binding of Tumor Necrosis Factor α (TNF-α) to its receptors [7].

## References

[1] Marfella R., di Filippo C., Portoghese M. (2009). The ubiquiti–proteasome system contributes to the inflammatory injury in ischemic diabetic myocardium: The role of glycemic control. Cardiovasc Pathol..

[2] Chen F.T., Liu Y.C., Yang C.M., Yang C.H. (2012). Anti-inflammatory effect of the proteasome inhibitor bortezomib on endotoxin-induced uveitis in rats. Investig. Ophthalmol. Vis. Sci..

[3] Hwee D.T., Gomes A.V., Bodine S.C. (2011). Cardiac proteasome activity in muscle ring finger-1 null mice at rest and following synthetic glucocorticoid treatment. Am. J. Physiol. Endocrinol. Metab..

[4] Jabs D.A., Rosenbaum J.T., Foster C.S., Holland G.N., Jaffe G.J., Louie J.S., Nussenblatt R.B., Stiehm E.R., Tessler H., van Gelder R.N. (2000). Guidelines for the use of immunosuppressive drugs in patients with ocular inflammatory disorders: Recommendations of an expert panel. Am. J. Ophthalmol..

[5] Nussenblatt R.B., Fortin E., Schiffman R., Rizzo L., Smith J., van Veldhuisen P., Sran P., Yaffe A., Goldman C.K., Waldmann T.A. (1999). Treatment of non-infectious intermediate and posterior uveitis with the humanized anti-Tac mAb: A phase I/II clinical trial. Proc. Natl. Acad. Sci. USA.

[6] Kotter I., Zierhut M., Eckstein A., Vonthein R., Ness T., Günaydin I., Grimbacher B., Blaschke S., Meyer-Riemann W., Peter H.H. (2003). Human recombinant interferon-α2a (rhIFN α2a) for the treatment of Behçet’s disease with sight-threatening retinal vasculitis. Adv. Exp. Med. Biol..

[7] Hale S., Lightman S. (2006). Anti-TNF therapies in the management of acute and chronic uveitis. Cytokine.

